# Comparison of Data Fusion Methods as Consensus Scores for Ensemble Docking

**DOI:** 10.3390/molecules24152690

**Published:** 2019-07-24

**Authors:** Dávid Bajusz, Anita Rácz, Károly Héberger

**Affiliations:** 1 Medicinal Chemistry Research Group, Research Centre for Natural Sciences, Hungarian Academy of Sciences, Magyar tudósok krt. 2, H-1117 Budapest, Hungary; 2 Plasma Chemistry Research Group, Research Centre for Natural Sciences, Hungarian Academy of Sciences, Magyar tudósok krt. 2, H-1117 Budapest, Hungary

**Keywords:** ensemble docking, data fusion, SRD, ROC curve, AUC, BEDROC

## Abstract

Ensemble docking is a widely applied concept in structure-based virtual screening—to at least partly account for protein flexibility—usually granting a significant performance gain at a modest cost of speed. From the individual, single-structure docking scores, a consensus score needs to be produced by data fusion: this is usually done by taking the best docking score from the available pool (in most cases— and in this study as well—this is the minimum score). Nonetheless, there are a number of other fusion rules that can be applied. We report here the results of a detailed statistical comparison of seven fusion rules for ensemble docking, on five case studies of current drug targets, based on four performance metrics. Sevenfold cross-validation and variance analysis (ANOVA) allowed us to highlight the best fusion rules. The results are presented in bubble plots, to unite the four performance metrics into a single, comprehensive image. Notably, we suggest the use of the geometric and harmonic means as better alternatives to the generally applied minimum fusion rule.

## 1. Introduction

Molecular docking has proven to be an invaluable tool of structure-based drug discovery in the past three decades, powering many virtual screening campaigns [1], which have resulted in the discovery of new kinase inhibitors [2], G-protein coupled receptor (GPCR) agonists and antagonists [3] and various other types of small-molecule ligands. Docking embodies a delicate balance between speed and precision: despite being based mostly on principles of molecular mechanics (and often involving a thorough evaluation of various intermolecular interaction terms), it is still capable of examining large ligand sets (up to the order of 10^6^ ligands) in a reasonable time. Hence, ligand docking lies in-between the fields of molecular modelling and cheminformatics. The rigid treatment of the protein structure allows for fast calculations, but it is also the greatest limitation of docking in terms of its precision, particularly for scoring. There are various approaches to at least partly increase precision at the cost of speed: the most popular one is undoubtedly ensemble docking, i.e., the simultaneous application of multiple, conformationally diverse structures to represent the protein [4]. The term “ensemble docking” was coined in the 2007 work of Huang and Zou [5], and methodologies for ensemble selection/optimization were proposed in the recent years [6,7], including cases of multiple experimental structures [8], as well as homology models [9]. By the present day, with the increase of computational power, ensemble docking has become a routine approach in early-stage drug discovery. While the current standards of ensemble docking rely on structure generation by molecular dynamics (MD) simulations and ensemble selection by the conformational clustering of (either experimental, or MD-generated) structures, researchers of the field are simultaneously exploring new directions for the enhanced sampling of conformational space, such as Markov state models [4].

From a molecular modelling perspective, many important methodological questions regarding ensemble docking have been studied, such as ensemble size, selection algorithm and other factors [10] and best practices were suggested for ensemble generation with molecular dynamics simulations [11] and normal mode analysis [12]. The effect of dataset composition on virtual screening was studied by Bojarski et al. [13,14], while various aspects of decoy selection were summarized in the recent review of Réau et al. [15]. However, if we look at ensemble docking from a cheminformatics point of view, it evidently presents a task of data fusion, i.e., how do we rank or select from the screened compounds when we have multiple docking score values for each of them? (More technically: how do we define the consensus docking score for a compound, given *n* docking scores on the individual protein structures?) While several data fusion methods exist – and have been thoroughly studied for ligand-based virtual screening [16,17] –, an extensive study to explore their application for ensemble docking is still lacking.

With this study, we aim to fill this void, by conducting a thorough statistical comparison of several data fusion options on multiple datasets, coming from ensemble docking-based virtual screenings on kinases (case studies 1 and 2) [18,19], a GPCR (G-protein coupled receptor) target (case study 3) [20], an oxidoreductase enzyme (case study 4), and a nuclear receptor (case study 5). Data fusion methods compiled by Willett are evaluated, including minimum, maximum, various types of averages and the Euclidean fusion rule [16]. A combination of performance metrics is applied for the evaluation, including area under the receiver operating characteristic (ROC) curve (AUC), average precision (AP), Boltzmann-enhanced discrimination of ROC (BEDROC) and sum of ranking differences (SRD) values. Ultimately, the paper intends to point the attention of the cheminformatics and drug discovery communities to the possible applications of data fusion rules in the context of ensemble docking.

## 2. Results and Discussion

Five case studies were applied for the statistical comparisons conducted in this work. Case study 1 was a virtual screening that has resulted in the identification of five new, potent inhibitors of Janus kinase 1 (JAK1, a kinase enzyme involved in autoimmune in inflammatory diseases) [18]. Likewise, case study 2 resulted in the discovery of six new inhibitors of JAK2 (subtype 2 of the same enzyme family) [19]. Both studies have employed ensemble docking into five protein structures (either a crystal structure, or selected from a molecular dynamics simulation) and involved a thorough optimization of the virtual screening workflows by retrospective virtual screening.

Case study 3 is a molecular modeling study focused on identifying the most important structural features for ligand recognition by the serotonin receptor 5-HT_6_, involving homology modeling and molecular dynamics (MD) simulations of the receptor [20]. To that end, the authors evaluated a large number of models and MD snapshots in retrospective virtual screening experiments, using three ligand sets from different sources. They identified nine protein structures that gave the best enrichment of active molecules in retrospective screening.

Case studies 4 and 5 are two retrospective screens conducted by us on the aldose reductase 2 enzyme (ALR2) and the estrogen receptor (ER), respectively, to provide a more diverse set of target proteins and allowing for more general conclusions. The ligand sets were downloaded from the DUD database [21], and the protein structures used for docking were selected by clustering from the available structures deposited in the Protein Data Bank (PDB) database.

From all of the five case studies, datasets used for retrospective screening (with docking scores and known active/inactive labels for each compound) were applied in the present work. Dataset compositions are summarized in Section 3.1.

Various data fusion rules were explored, as collected and detailed by Willett [16]; these included the minimum (MIN) and maximum (MAX) rules—which simply assign the smallest (best) and largest (worst) individual docking score as the consensus score for each compound—, the mean (SUM), median (MED) and Euclidean norm (EUC) rules, and two further types of average: the geometric (GEOM) and harmonic means (HARM). The formulas are summarized in Section 3.2. Originally, the reciprocal rank fusion (RRF) rule was considered as well, but it quickly became clear that without setting a threshold (e.g., considering only the top 1 % of each list of docking scores) it provides highly redundant values (see Supplementary Figure S1a,b), thus it was omitted from further study.

**Figure 1:**
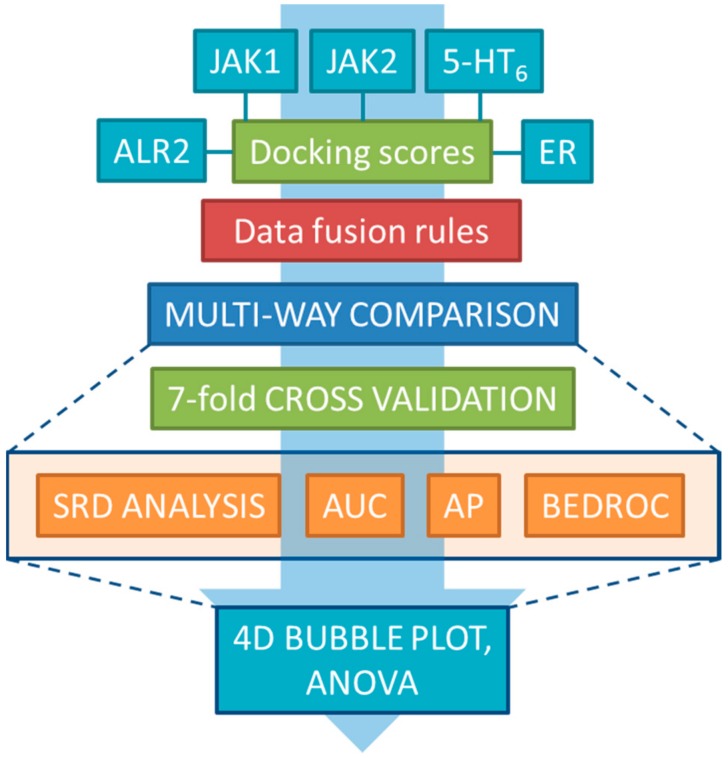
The workflow of the complete study. Single-structure docking scores from five case studies were used to generate consensus scores by the various data fusion rules, which were then compared by four performance metrics (SRD, AUC, AP, BEDROC) and statistical analysis (ANOVA).

For the statistical comparison of the fusion rules, we have applied several performance metrics, including the area under the ROC curve values (AUC), average precision (AP), Boltzmann-enhanced discrimination of receiver operating characteristic (BEDROC) and sum of ranking differences (SRD)—these are summarized in Section 3.3. The workflow of the project is included in Figure 1.

**Figure 2:**
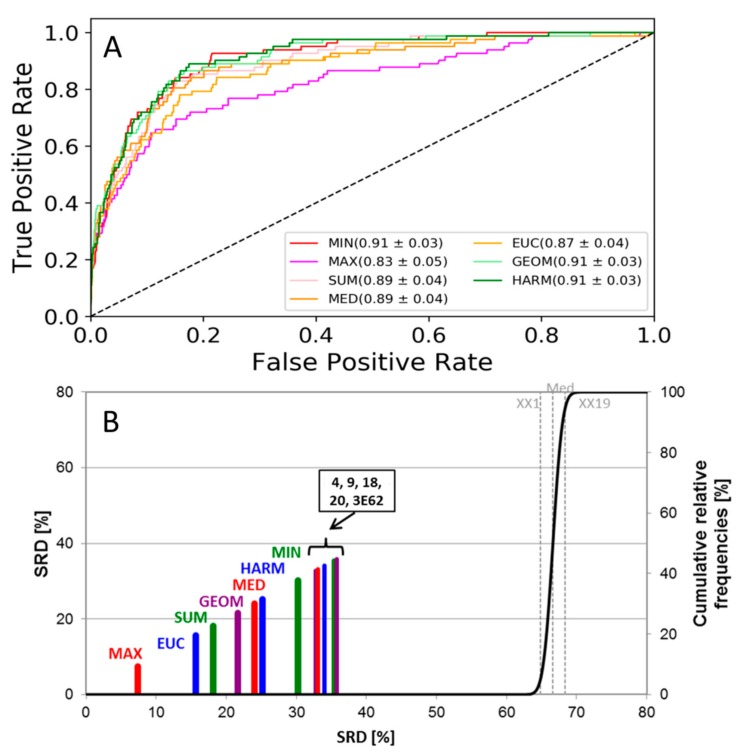
(**A**) ROC curves of the JAK2 dataset. The AUC values and standard deviations for each fusion rule are included in the legend. Single-structure docking scores are omitted for clarity. (**B**) SRD analysis of the JAK2 dataset. Normalized SRD values are plotted on the x and left y axes, and the cumulative relative frequencies of SRD values for random ranking are plotted on the right y axis and shown as the black curve. Single-structure docking scores are labeled based on PDB code (3E62) or sequential MD frame number (4, 9, 18, 20).

For the JAK1 and JAK2 datasets, as a first level of comparison, we have calculated ROC curves and the corresponding AUC values (standard deviations were calculated based on sevenfold cross-validation). ROC curves for the JAK2 dataset are shown in Figure 2A (and Supplementary Figure S2 for JAK1, with highly similar results).

As seen in Figure 2A, ROC curves provide little distinction between the data fusion rules, although it is clear that the maximum rule (MAX) is somewhat worse than the others for both datasets. Since the maximum rule selects the worst docking score, we can conclude that it is not advantageous to apply the strictest possible fusion rule.

To provide a more detailed picture, we have extended the comparison to include other performance metrics as well. Average precision (AP) is calculated analogously to AUC, but instead of the ROC curve, the area under the precision-recall curve is calculated. Thus, AP gives information about how precise the selection of compounds is, which is the fraction of actives among the selected compounds averaged over the varying score thresholds *T*. BEDROC is a weighted version of the AUC value where the early part of the ROC curve is given more weight—meaning that BEDROC focuses more on early enrichment. Finally, sum of ranking differences (SRD) is a robust statistical method, specifically developed for method comparison tasks. Briefly, it evaluates Manhattan distances of a set of rank transformed vectors (here, corresponding to different data fusion rules) from a reference vector which corresponds to a hypothetical ideal reference method. The reference vector can be a “gold standard” or experimental values (where available), or a consensus method based on data fusion itself. In our case, an ideal method would produce the best (lowest) possible consensus scores for the active molecules, and the worst (highest) possible scores for the inactives; thus, we have defined the reference vector by taking the lowest single-structure docking score for the actives and the highest for the inactives (minimax rule). The performance metrics are explained in more detail in Section 3.3.

An extensive statistical analysis was carried out on the AUC, AP, BEDROC and SRD values for significance testing between the fusion rules with one-way ANOVA and Tukey HSD *post-hoc* tests. Even if the standard deviations have overlapped in several cases, the fusion rules have proven to be significant as the ANOVA factor, and Tukey HSD tests have shown the significant difference between most pairs of the fusion rules and single-structure docking scores. A brief summary of the Tukey HSD tests with the ratios of the significant and non-significant pairs of variables can be found in the Supplementary Materials (Figure S3a–c) for each dataset, while the ANOVA results are tabulated in Supplementary Table S1a–t.

The SRD plot is shown in Figure 2B for the JAK2 dataset and in Supplementary Figure S4 for the JAK1 dataset, which yielded similar results. Sevenfold randomized cross-validation was used for establishing uncertainty to SRD values, and normalized SRD values were used for comparability. All the data fusion methods and single-structure docking scores were better than random ranking, as they do not overlap with the cumulative relative frequency curve of random ranking. Based on SRD, all fusion rules are more consistent with the reference than single-structure docking scores (4, 9, 18, 20, 3E62), which was not unexpected—except for MIN (in the JAK2 dataset) and MAX (in the JAK1 dataset). The various types of averages and the Euclidean norm are consistently among the best options.

The same workflow was applied to test the fusion rules for the ensemble docking of a dataset of 5-HT_6_ ligands (case study 3), based on docking to the nine best receptor structures that were identified in the respective study [20].

**Figure 3:**
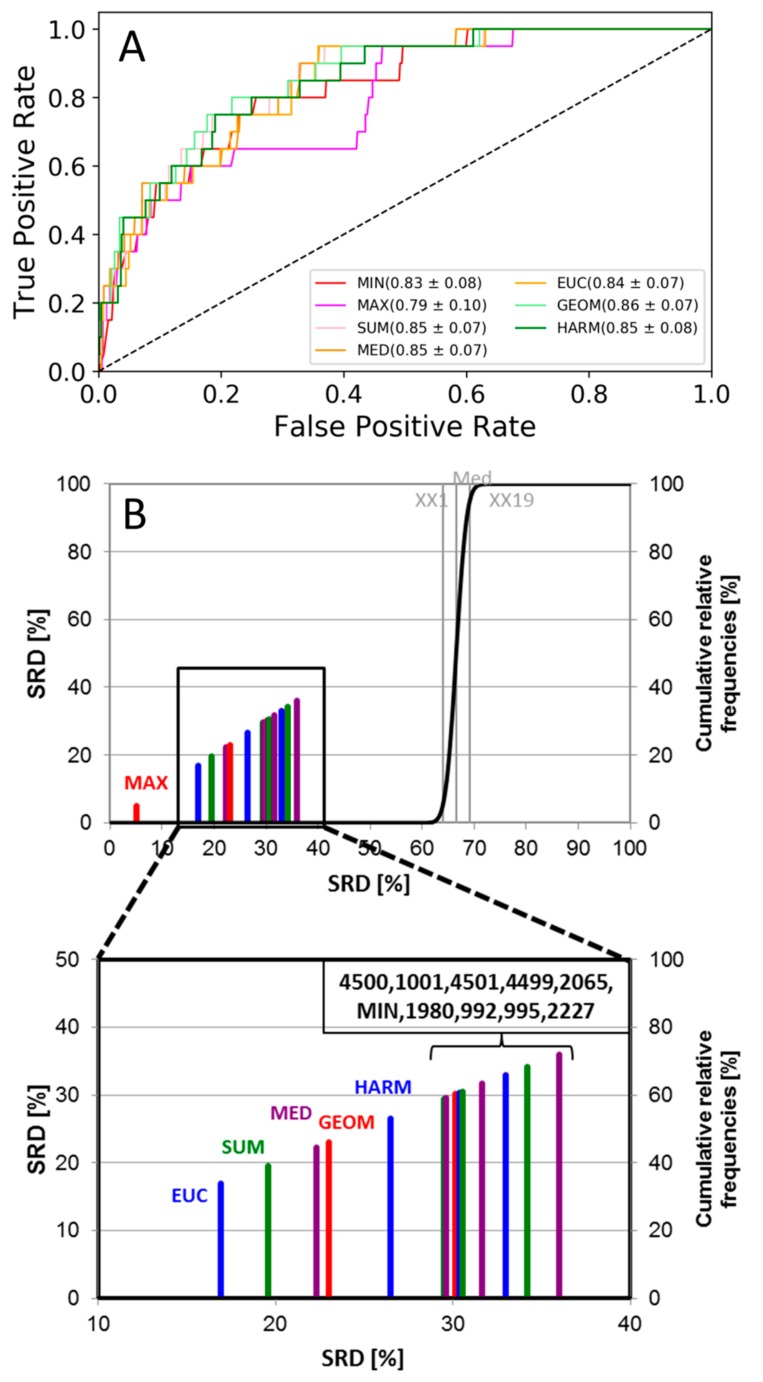
(**A**) ROC curves of the 5-HT_6_ dataset. The AUC values and standard deviations for each fusion rule are included in the legend. Single-structure docking scores are omitted for clarity. (**B**) SRD analysis of the 5-HT6 dataset. Normalized SRD values are plotted on the x and left y axes, and the cumulative relative frequencies of SRD values for random ranking are plotted on the right y axis and shown as the black curve. The MD frame numbers in the bracket denote single-structure docking scores; the structure labels are taken from Table 2 of case study 3 [20].

ROC curves of the fusion rules are plotted in Figure 3A. Similarly to case studies 1 and 2, only the maximum rule stands out as a somewhat worse option as compared to the others, but all of them are far from random. Nonetheless, we wanted to see whether the four performance metrics could distinguish between the fusion rules in this setting; therefore, we have carried out the same analysis as for case studies 1 and 2.

According to the SRD result (Figure 3B), the various averages and the maximum rule (MAX) were closest to the ideal reference vector, while the minimum rule (MIN) overlapped with the single-structure docking scores. All the fusion rules and docking scores were better than random ranking. All of the four performance metrics were checked with ANOVA as well, and the difference between the data fusion methods were significant (at α = 0.05) in each case.

**Figure 4:**
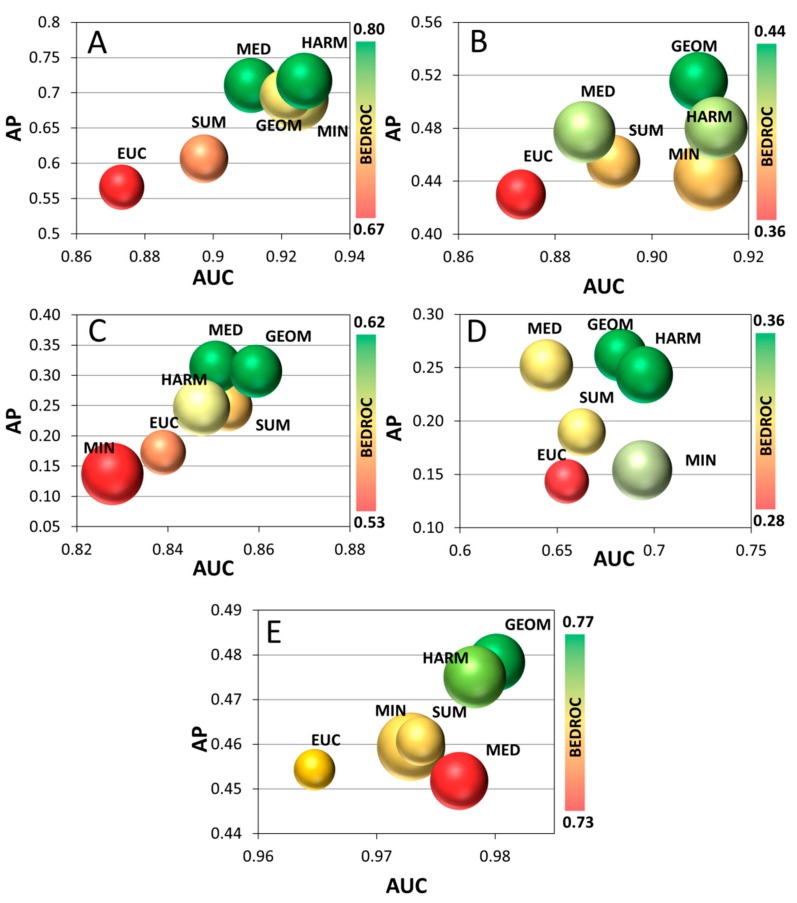
Bubble plots of the JAK1 (**A**), JAK2 (**B**), 5-HT6 (**C**), ALR2 (**D**) and ER (**E**) datasets. AP values are plotted against the AUC values. Bubble sizes correspond to SRD values (the smaller the better) and the colors correspond to BEDROC values, increasing from red to green (see color scale on the right). The MAX rule and single-structure docking scores were omitted due to their greater distance from the other fusion rules (see Supplementary Figure S5).

To summarize the four performance metrics in a single image, 4D bubble plots were created (Figure 4). AUC and AP values were plotted on the x and y axis, respectively, SRD values were used as the size of the bubbles (the smaller the better), and the colors were assigned based on the BEDROC values (increasing from red to green). The plotted values are the average of every cross-validation section for all four metrics. Single-structure docking scores and the maximum rule (MAX) were omitted from this figure due to the bigger distance from the others; the omitted ones are compared with the best method in Supplementary Figure S5.

To extend the scope of our study to more protein targets, two further case studies were conducted on a non-kinase enzyme (aldose reductase 2, case study 4) and a non-GPCR receptor (estrogen receptor, case study 5), respectively. In these case studies, protein structures were selected by clustering the available experimental structures from the PDB database and selecting the centroids for each cluster (see Section 3.1 for more detail). The results of these case studies are presented as 4D bubble plots in Figure 4 as well.

Capturing all of the available information in a single plot provides a clearer distinction of the data fusion rules. First, it is evident that the strictest fusion rule (MAX) falls short of the rest of the options. Also, while there is a general trend of the various performance metrics changing in a concordant way; e.g., AP increasing together with AUC, some exceptions arise. Most notably, the *de facto* standard minimum fusion rule (MIN), which boasts good AUC values, is relatively inconsistent with regard to the rest of the three performance metrics. Results for the median (MED) rule are mostly promising. However, while it seems to be among the best fusion rules for the JAK1 and 5-HT6 datasets, its BEDROC, AP and AUC values are significantly worse in case studies 4 and 5. On the other hand, the geometric mean (GEOM) and harmonic mean (HARM) are consistently among the best options based on all of the performance metrics. They both provide good overall retrieval of actives (AUC), as well as early enrichment (BEDROC) and consistence with the ideal reference method (SRD) for all datasets. Therefore, based on the results on these five case studies, we can recommend the geometric and harmonic means as new fusion rules for creating a consensus score after ensemble docking.

Maximum (MAX), again, overlapped with the single-structure docking scores, and therefore it was omitted from Figure 4 (see Supplementary Figure S5). The results confirm the main conclusions drawn from the first two case studies, with the cluster comprising the various averages and the median forming the best options. In comparison, the two most consistent options are the geometric (GEOM) and harmonic means (HARM), followed by the median (MED). minimum (MIN) proves to be a suboptimal solution here, whereas the arithmetic mean (SUM) and the Euclidean distance (EUC) are somewhat inconsistent among the five case studies.

## 3. Methods

### 3.1. Dataset Generation and Docking

Five case studies were used for the evaluations. Case studies 1 and 2 were virtual screenings that resulted in the identification of five and six new, potent inhibitors of Janus kinase subtypes 1 (JAK1) [18] and 2 (JAK2) [19], respectively. Janus kinases are a family of tyrosine protein kinases involved in the JAK/STAT intracellular signaling pathway, with pharmacological relevance in a number of autoimmune and inflammatory diseases [22]. In both studies, a thorough retrospective screening, based on known literature inhibitors and a decoy set from the DUD database [21], was carried out to optimize the virtual screening protocol and compile the protein structure ensemble from a pool of available crystal structures and molecular dynamics (MD) simulation frames. Active compounds—reported IC_50_ below 1 μM—and their bioactivity data were manually collected from numerous literature sources, while the decoy set for CDK2—the closest kinase based on sequence identity, with a decoy set available at the time—was downloaded from the DUD database. Briefly, ligand preparation and docking were carried out with Schrödinger’s Ligprep and Glide programs [23], respectively. Dataset compositions are included in Table 1, the virtual screening protocols are presented in more detail in refs. [18,19].

Case study 3 presented a thorough molecular modeling study, involving homology modeling and molecular dynamics (MD) simulations of the 5-HT_6_ receptor [20]. The 5-HT_6_ receptor is a G-protein coupled serotonin receptor with great potential for the treatment of neuropathological disorders, such as Parkinson’s disease, Alzheimer’s disease, and schizophrenia [24]. Homology modeling was carried out with Schrödinger’s Prime [25], while the MD simulations were conducted with the NAMD software [26]. Three ligand sets were used to evaluate a large number of models and MD snapshots, to determine the most important structural determinants of ligand recognition by the receptor. Here, we apply the docking scores that resulted for the dataset of 5-HT_6_ antagonists (K_i_ < 100 nM) and experimentally confirmed inactives included in the Drugmatrix dataset, which were hosted on the ChEMBL database [27]. Table 2 of the paper identifies nine specific structures from MD simulations that were particularly successful in retrospective virtual screening tests. Docking results on these nine structures for the Drugmatrix set [27] were applied in the present study for the comparison of the data fusion methods.

To allow for more general conclusions on a more diverse set of protein targets, two further datasets were downloaded from the directory of useful decoys (DUD) [21]: Case studies 4 and 5 are two retrospective virtual screens that we conducted against aldose reductase 2 (ALR2), and the estrogen receptor (ER), respectively. Aldose reductase 2 is a key enzyme in the polyol pathway: it catalyzes the reduction of glucose to sorbitol, and is also involved in the development of cardiovascular complications in diabetes mellitus [28]. The discovery of ALR2 inhibitors are therefore highly desired, to prevent the onset of these complications (such as ischemia, atherosclerosis or atherothrombosis). Estrogen has a wide role in human physiology, including, among others, reproduction, cardiovascular health, cognition and behavior [29]. As a consequence, it is implicated in the development of a number of diseases, from neurodegenerative diseases to obesity. Since estrogen mediates its effects through the estrogen receptor, this target is the basis for therapeutic intervention.

Table 1 summarizes the dataset compositions of the five case studies, in terms of the number of active and inactive molecules, the number and type (X-ray, MD) of protein structures, and the starting structures for the MD simulations, where applicable. RMSD values between the protein structures are included in Supplementary Table S2. All protein structures used for docking or MD simulations, including the homology model template 5-HT_2B_ structure in case study 3, were ligand-bound. The ensemble selection approach for case studies 1 and 2 is published in detail in [18], where the ensembles were iteratively optimized to produce the best early enrichments on the training datasets. In case study 3, the nine best structures were selected based on their individual virtual screening performances [20]. In case studies 4 and 5, the X-ray structures available in the PDB database (141 and 251, respectively) were clustered, based on a complete RMSD matrix calculated on the binding site residues, using the Euclidean distance, the complete linkage method and distance cutoffs of 1 Å and 4 Å, respectively. The centroid of the resulting six, and three clusters were selected.

### 3.2. Data Fusion Methods

Data fusion rules for similarity rankings employed in ligand-based virtual screening were collected and summarized in the work of Willett [16]. These include the simple minimum and maximum (MIN and MAX) rules, as well as the arithmetic (SUM), geometric (GEOM) and harmonic means (HARM), the median (MED), and the Euclidean norm (EUC), which views the set of (docking) scores for each compound as an *n*-dimensional vector and computes the Euclidean norm for that vector. Since we wanted to apply the fusion rules to the whole datasets, we have discarded the cutoff-based ANZ and MNZ rules. Also, while we have originally considered the reciprocal rank fusion (RRF) rule as well, it quickly became apparent that it produces noisy and mostly random numbers for larger datasets without setting a cut-off (i.e., considering the whole dataset rather than only the top *x %*, see Supplementary Figure S1a,b). The formulas of the fusion rules are summarized in Table 2.

### 3.3. Performance Metrics and Statistical Analysis

The different data fusion methods were compared by several performance metrics. In this sense, it can be considered as multicriteria optimization as well. These include “global” performance metrics, such as the area under the (ROC) curve (AUC) and average precision (AP), the Boltzmann-enhanced discrimination of receiver operating characteristic—BEDROC, which focuses more on early enrichment—, and a robust statistical tool for method comparison, sum of ranking differences (SRD).

For classification by docking results, we usually need to define a score threshold *T* to assign which molecules are predicted to be actives (docking score better than or equal to *T*) and inactives (docking score worse than *T*). Then, if the true class labels are known (retrospective screening), we can determine the number of true positives (*TP*), false positives (*FP*), true negatives (*TN*) and false negatives (*FN*) and many derived parameters, such as the true positive rate or recall:

(1) $$\mathrm{TPR}=\frac{TP}{TP+FN}\;,$$

the false positive rate:

(2) $$\mathrm{FPR}=\frac{FP}{FP+TN}\;,$$

or the precision:

(3) $$\mathrm{Pr}=\frac{TP}{TP+FP}\;.$$

**Figure 5:**
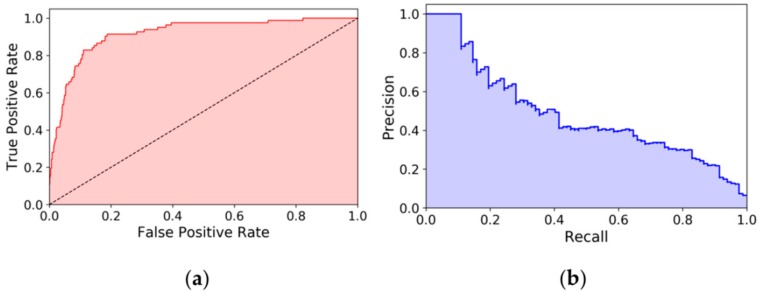
Example for (**a**) a ROC plot, and (**b**) a precision-recall curve (consensus score with the MIN fusion rule on the JAK2 dataset). The areas under the curves are (**a**) the AUC value (here, 0.916), and (**b**) the AP (average precision) value (here, 0.484), respectively. The dashed line on the ROC plot corresponds to random classification.

However, setting the threshold *T* involves an inevitable level of subjectivity. The advantage of “global” performance parameters such as AUC and average precision is that they eliminate this subjectivity by accounting for all possible threshold values and compacting this information into a single number. AUC is calculated by plotting the true positive rates against the false positive rates (by increasing score thresholds *T*) and taking the area under the curve, while average precision (AP) is calculated analogously from the precision-recall plot (Figure 5). The BEDROC metric was proposed by Truchon and Bayly to tackle the “early recognition” problem by using a continuously decreasing exponential weight as a function of rank [43]. Briefly, BEDROC can be considered as a weighted version of the AUC value, where the beginning of the ROC curve is taken into account with a greater weight. Thus, BEDROC values reflect early enrichment rather than the “global” performance of the classifier. AUC, AP and BEDROC values—the latter with an α = 20 parameter setting—were calculated with the Scikit-learn Python package [44].

Sum of ranking differences (SRD) can be applied for the comparison of different metrics, models, methods etc., and in our case: the data fusion methods [45,46]. The method is robust, but also sensitive to small differences between the compared variables. By convention, the data matrix contains the comparable variables (data fusion methods) in the columns and the samples (molecules) in the rows. It is crucial that a reference vector is added as the last column of the matrix, which can be a set of known reference values, e.g., from a measurement if available, but we can also use row-wise data fusion (average, minimum or maximum), if an exact reference vector is not available. Data fusion is an inherent, crucial step in SRD analysis. Row-maximums are selected as a gold standard for those magnitudes where the ‘larger the better’ reasoning applies (e.g., for correlation coefficients, accuracy, etc.) and row minimums for those magnitudes where the ‘smaller the better’ applies (RMSE, misclassification errors, etc.) With this approach we can hypothetically define the best method that unites all the advantageous features. Then, the variables—including the reference vector—are rank transformed in increasing magnitude. Then, the absolute differences between the ranks assigned by the reference vector and the other variables are calculated one-by-one, for each sample and each variable. In the final step, these differences are summed for each variable (i.e., column-wise), which gives the SRD values. SRD values represent a Manhattan distance from the reference vector (commonly normalized between 0 and 100); thus, the smaller the better. The concept of SRD is illustrated in more detail in our recent work [47]. SRD has two types of validation protocol: cross-validation and a randomization test. With the randomization test, we can establish whether the ranking produced by a variable/method (here, data fusion rule) is significantly different from assigning ranks randomly, whereas with cross-validation we can test whether the SRD value distributions of two methods (fusion rules) are significantly different. SRD applies special plots, where the column heights do not carry information, but fairly the proximity of the columns to zero and to the frequency distribution curve of random ranking. We have used SRD in our recent works for the comparison of similarity metrics for molecular [47], metabolomic [48] and interaction fingerprints [49].

In the present study, the single-structure docking scores were rank transformed prior to computing the reference vector to remove any docking score offset between the various protein structures, and the resulting datasets were standardized for the SRD analysis itself, as the data fusion metrics produce values in different ranges. Sevenfold cross-validation with randomized selection was applied for the validation procedure. The reference column was defined in the following way: for the active molecules, the row-minima were used, and for the decoy molecules, the row-maxima were used. (A hypothetical, ideal reference method should provide the lowest, i.e., best possible score for actives and the highest, i.e., worst score for inactives.)

Multiway comparison was based on the average values of the results of cross-validations for each metric (SRD, AUC, AP, BEDROC). The average values were compared in 4D bubble plots. The coloring scheme for the bubble plot was based on the BEDROC values (increasing from red to green), while bubble sizes were based on the SRD values (the smaller the better).

One-way ANOVA was also used to test the significance of the different data fusion methods (as factor) based on the above mentioned four parameters.

## 4. Conclusions

Seven different fusion rules were compared in ensemble docking studies with a multi-way comparison protocol to find the best and most consistent options to create consensus scores from the single-structure docking scores. To that end, three case studies reporting retrospective virtual screenings were collected from the literature, corresponding to two major classes of currently investigated drug targets (kinases and GPCRs). Two further retrospective virtual screens were conducted as part of the present study on two further, relevant drug targets. Four different performance metrics: area under the ROC curve (AUC), average precision (AP), Boltzmann-enhanced discrimination of ROC (BEDROC) and sum of ranking differences (SRD) values were used in the comparison to find the most optimal solution. We can conclude that the use of data fusion methods have statistically significant effects on the outcome. The typical solution with the use of the minimum score as the fusion rule (MIN) was not the best option according to the four performance metrics. It was surpassed by the geometric (GEOM) and harmonic means (HARM), which clearly outperformed the others. On the other end of the scale, the strictest maximum (MAX) fusion rule is not recommended. The two methods mentioned above could provide consistently better results based on the multi-way comparison and are hereby suggested for the drug discovery community for use in ensemble docking studies.

## Figures and Tables

**Table 1:** Dataset composition of the five case studies.

#	Dataset Name	Protein Structures			Actives	Inactives
		X-ray	MD Frames	MD Starting Structure		
1	JAK1	3EYG [30], 4IVC [31]	3	4IVC [31]	115	1644
2	JAK2	3E62 [32]	4	3TJD [33]	82	1437
3	5-HT_6_	-	9	homology model based on 4IB4 [34]	20	689
4	ALR2	1IEI [35], 2FZD [36], 2PFH [37], 3LZ5 [38], 3M0I [39], 4GCA	-	-	26	917
5	ER	2BJ4 [40], 5TM9 [41], 6CHZ [42]	-	-	38	1344

**Table 2:** Formulas of the applied fusion rules.

Rule	Full Name	Formula^1^
MIN	Minimum	min(DS_1_, DS_2_ … DS_n_)
MAX	Maximum	max(DS_1_, DS_2_ … DS_n_)
SUM	Arithmetic mean	$\frac{1}{n}\sum_{i=1}^{n}DS_{i}$
GEOM	Geometric mean	$\sqrt[{n}]{\prod_{i=1}^{n}DS_{i}}$
HARM	Harmonic mean	${\left(\frac{\sum_{i=1}^{n}DS_{i}{}^{-1}}{n}\right)}^{-1}$
MED	Median	median(DS_1_, DS_2_ … DS_n_)
EUC	Euclidean norm	$\sqrt{\sum_{i=1}^{n}DS_{i}{}^{2}}$

^1^ Docking scores are denoted by DS_1_, DS_2_ … DS_n_.

## Supplementary Materials

The following are available online, Supplementary Figures S1–S5, Supplementary Table S1.
