# Supplementary material for: Comparison of Data Fusion Methods as Consensus Scores for Ensemble Docking
Source: Molecules. 2019 Jul 24;24(15):2690. doi: 10.3390/molecules24152690 (PMC6695709; doi:10.3390/molecules24152690)
Supplement: Supplementary file 1 [file molecules-24-02690-s001.pdf]

Supplementary information

Comparison of data fusion methods for ensemble docking

Dávid Bajusz[1], Anita Rácz[2,*], Károly Héberger[2]

[1] Medicinal Chemistry Research Group, Research Centre for Natural Sciences, Hungarian Academy of Sciences, H-1117 Budapest, Magyar tudósok krt. 2, Hungary

[2] Plasma Chemistry Research Group, Research Centre for Natural Sciences, Hungarian Academy of Sciences, H-1117 Budapest, Magyar tudósok krt. 2, Hungary

*Correspondence: racz.anita@ttk.mta.hu

Table S1a-t. Summary of ANOVA results for the five datasets. The „Fusion rule" factor is always significant at a level of α = 0.05.

a) JAK1 – AP - ANOVA

Effect	Univariate Tests of Significance for AP – JAK1 dataset				
	SS	Degr. of Freedom	MS	F	p
Intercept	36.99393	1	36.99393	43952.15	0.00
Metric	0.49786	11	0.04526	53.77	0.00
Error	0.07070	84	0.00084		

b) JAK1 – AUC – ANOVA

Effect	Univariate Tests of Significance for AUC – JAK1 dataset				
	SS	Degr. of Freedom	MS	F	p
Intercept	74.68951	1	74.68951	1220250	0.00
Metric	0.18780	11	0.01707	279	0.00
Error	0.00514	84	0.00006		

c) JAK1 – BEDROC – ANOVA

Effect	Univariate Tests of Significance for BEDROC – JAK1 dataset				
	SS	Degr. of Freedom	MS	F	p
Intercept	49.77798	1	49.77798	264769.3	0.00
Metric	0.33709	11	0.03064	163.0	0.00
Error	0.01579	84	0.00019		

d) JAK1 – SRD – ANOVA

Effect	Univariate Tests of Significance for SRD – JAK1 dataset				
	SS	Degr. of Freedom	MS	F	p

Intercept	83557.16	1	83557.16	278889.3	0.00
DF	8879.10	11	807.19	2694.2	0.00
Error	46.74	84	0.30		

e) JAK2 – AP – ANOVA

Effect	Univariate Tests of Significance for AP – JAK2 dataset				
	SS	Degr. of Freedom	MS	F	p
Intercept	16.97439	1	16.97439	27085.71	0.00
Metric	0.33239	11	0.03022	48.22	0.00
Error	0.05264	84	0.00063		

f) JAK2 – AUC – ANOVA

Effect	Univariate Tests of Significance for AUC – JAK2 dataset				
	SS	Degr. of Freedom	MS	F	p
Intercept	72.04237	1	72.04237	1611050	0.00
Metric	0.11786	11	0.01071	240	0.00
Error	0.00376	84	0.00004		

g) JAK2 – BEDROC – ANOVA

Effect	Univariate Tests of Significance for BEDROC – JAK2 dataset				
	SS	Degr. of Freedom	MS	F	p
Intercept	29.28167	1	29.28167	57135.06	0.00
Metric	0.21825	11	0.01984	38.71	0.00
Error	0.04305	84	0.00051		

h) JAK2 – SRD – ANOVA

Effect	Univariate Tests of Significance for SRD – JAK2 dataset				
	SS	Degr. of Freedom	MS	F	p
Intercept	57350.45	1	57350.45	143964.8	0.00
DF	6308.61	11	573.51	1439.7	0.00
Error	28.68	84	0.40		

i) 5-HT6 – AP – ANOVA

Effect	Univariate Tests of Significance for AP – 5-HT6 dataset				
	SS	Degr. of Freedom	MS	F	p
Intercept	5.137960	1	5.137960	7889.409	0.00
Metric	0.545613	15	0.036374	55.853	0.00
Error	0.072940	112	0.000651		

j) 5-HT6 – AUC – ANOVA

Effect	Univariate Tests of Significance for AUC – 5-HT6 dataset				
	SS	Degr. of Freedom	MS	F	p
Intercept	85.57978	1	85.57978	328223.3	0.00
Metric	0.19218	15	0.01281	49.1	0.00
Error	0.02920	112	0.00026		

k) 5-HT6 – BEDROC – ANOVA

Effect	Univariate Tests of Significance for BEDROC – 5-HT6 dataset				
	SS	Degr. of Freedom	MS	F	p
Intercept	16.86602	1	16.86602	13397.69	0.00
Metric	0.45853	15	0.03057	24.28	0.00
Error	0.14099	112	0.00126		

l) 5-HT6 – SRD – ANOVA

Effect	Univariate Tests of Significance for SRD – 5-HT6 dataset				
	SS	Degr. of Freedom	MS	F	p
Intercept	223320.4	1	223320.4	26993.73	0.00
Metric	2700.8	15	180.1	21.76	0.00
Error	794.2	112	8.3		

m) ALR2 – AP – ANOVA

Effect	Univariate Tests of Significance for AP – ALR2 dataset				
	SS	Degr. of Freedom	MS	F	p
Intercept	3.841659	1	3.841659	4964.853	0.00
Metric	0.539073	12	0.044923	58.057	0.00
Error	0.070413	91	0.000774		

n) ALR2 – AUC – ANOVA

Effect	Univariate Tests of Significance for AUC – ALR2 dataset				
	SS	Degr. of Freedom	MS	F	p
Intercept	44.07959	1	44.07959	93997.99	0.00
Metric	0.25393	12	0.02116	45.12	0.00
Error	0.04267	91	0.00047		

o) ALR2 – BEDROC – ANOVA

Effect	Univariate Tests of Significance for BEDROC – ALR2 dataset				
	SS	Degr. of Freedom	MS	F	p
Intercept	9.541652	1	9.541652	12981.97	0.00
Metric	0.589185	12	0.049099	66.80	0.00
Error	0.066884	91	0.000735		

p) ALR2 – SRD – ANOVA

Effect	Univariate Tests of Significance for SRD – ALR2 dataset				
	SS	Degr. of Freedom	MS	F	p
Intercept	226349.6	1	226349.6	540639.5	0.00
Metric	19058.3	12	1588.2	3793.4	0.00
Error	70.8	91	0.4		

q) ER – AP – ANOVA

Effect	Univariate Tests of Significance for AP – ER dataset				
	SS	Degr. of Freedom	MS	F	p
Intercept	16.08263	1	16.08263	50828.70	0.00
Metric	0.10573	9	0.01175	37.13	0.00
Error	0.02215	70	0.00032		

r) ER – AUC – ANOVA

Effect	Univariate Tests of Significance for AUC – ER dataset				
	SS	Degr. of Freedom	MS	F	p
Intercept	74.59220	1	74.59220	2615563	0.00
Metric	0.02972	9	0.00330	116	0.00
Error	0.00200	70	0.00003		

s) ER – BEDROC – ANOVA

Effect	Univariate Tests of Significance for BEDROC – ER dataset				
	SS	Degr. of Freedom	MS	F	p
Intercept	42.94097	1	42.94097	222991.2	0.00
Metric	0.09498	9	0.01055	54.8	0.00
Error	0.01348	70	0.00019		

t) ER – SRD – ANOVA

Effect	Univariate Tests of Significance for SRD – ER dataset				
	SS	Degr. of Freedom	MS	F	p

**Intercept**	96563.91	1	96563.91	165669.3	0.00
**Metric**	13687.53	9	1520.84	2609.2	0.00
**Error**	75.77	70	0.58		

Table S2a-e. Root mean squared distances (RMSD, in Ångströms) between the (full) protein structures of the ensembles.

a) Case study 1 (structure numbers correspond to PDB code or nth nanosecond of the MD simulation)

	4IVC	5	9	13
3EYG	12.15	10.35	10.12	10.36
4IVC		7.58	7.84	7.77
5			3.95	2.34
9				3.99

b) Case study 2 (structure numbers correspond to PDB code or nth nanosecond of the MD simulation)

	9	18	20	3E62
4	2.19	3.47	2.45	10.1
9		3.69	2.39	10.33
18			3.58	9.82
20				10.13

c) Case study 3 (structure numbers correspond to MD frame numbers from Vass et al. *J. Comput. Aided. Mol. Des.* **2015**, *29*, 1137–1149.)

	995	1001	4499	4500	4501	1980	2065	2227
992	0.71	0.92	1.86	1.85	1.93	1.72	1.81	1.84
995		0.78	1.90	1.88	1.95	1.70	1.83	1.85
1001			1.96	1.93	2.01	1.78	1.89	1.90
4499				0.54	0.65	1.81	1.85	1.89
4500					0.68	1.84	1.89	1.91
4501						1.86	1.90	1.87
1980							0.96	1.21
2065								1.24

d) Case study 4 (structure numbers correspond to PDB codes)

	2FZD	2PFH	3LZ5	3M0I	4GCA
1IEI	3.57	3.61	3.55	3.56	0.67
2FZD		0.97	0.46	0.51	3.53
2PFH			0.66	0.86	3.58
3LZ5				0.45	3.51
3M0I					3.51

e) Case study 5 (structure numbers correspond to PDB codes)

	5TM9	6CHZ
2BJ4	7.54	7.64
5TM9		5.95

Figure S1ab. Comparison of the distributions of the RRF (**a**) and EUC (**b**) fusion rules based on the JAK1 dataset.

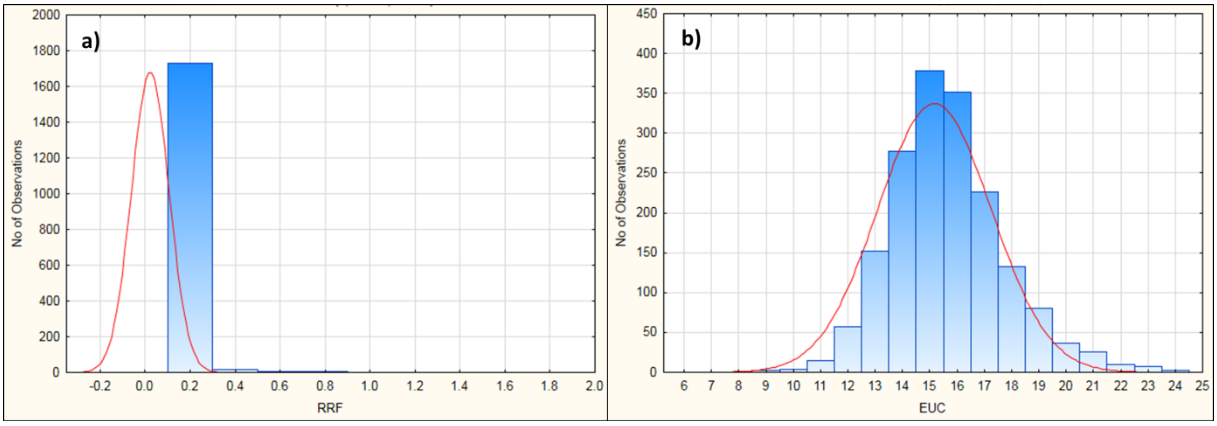

Figure S2. ROC curves of the JAK1 dataset. The AUC values and standard deviations for each method can be found in brackets.

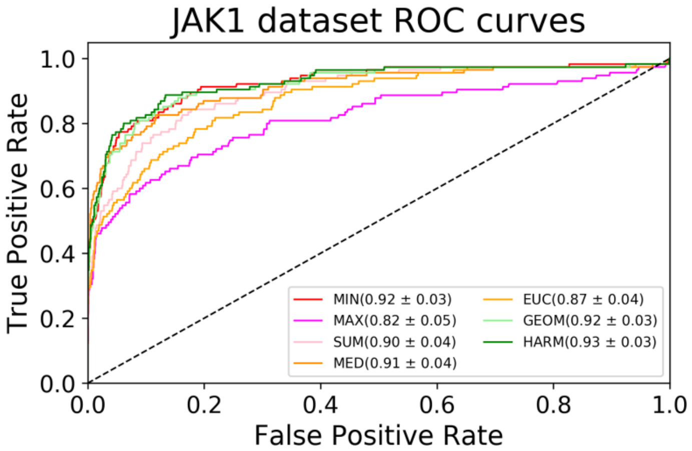

Figure S3a-e. Tukey HSD test results; the ratios of significant and non-significant pairs of variables (fusion rules or single structures) in the datasets are plotted on the barplots (α=0.05).

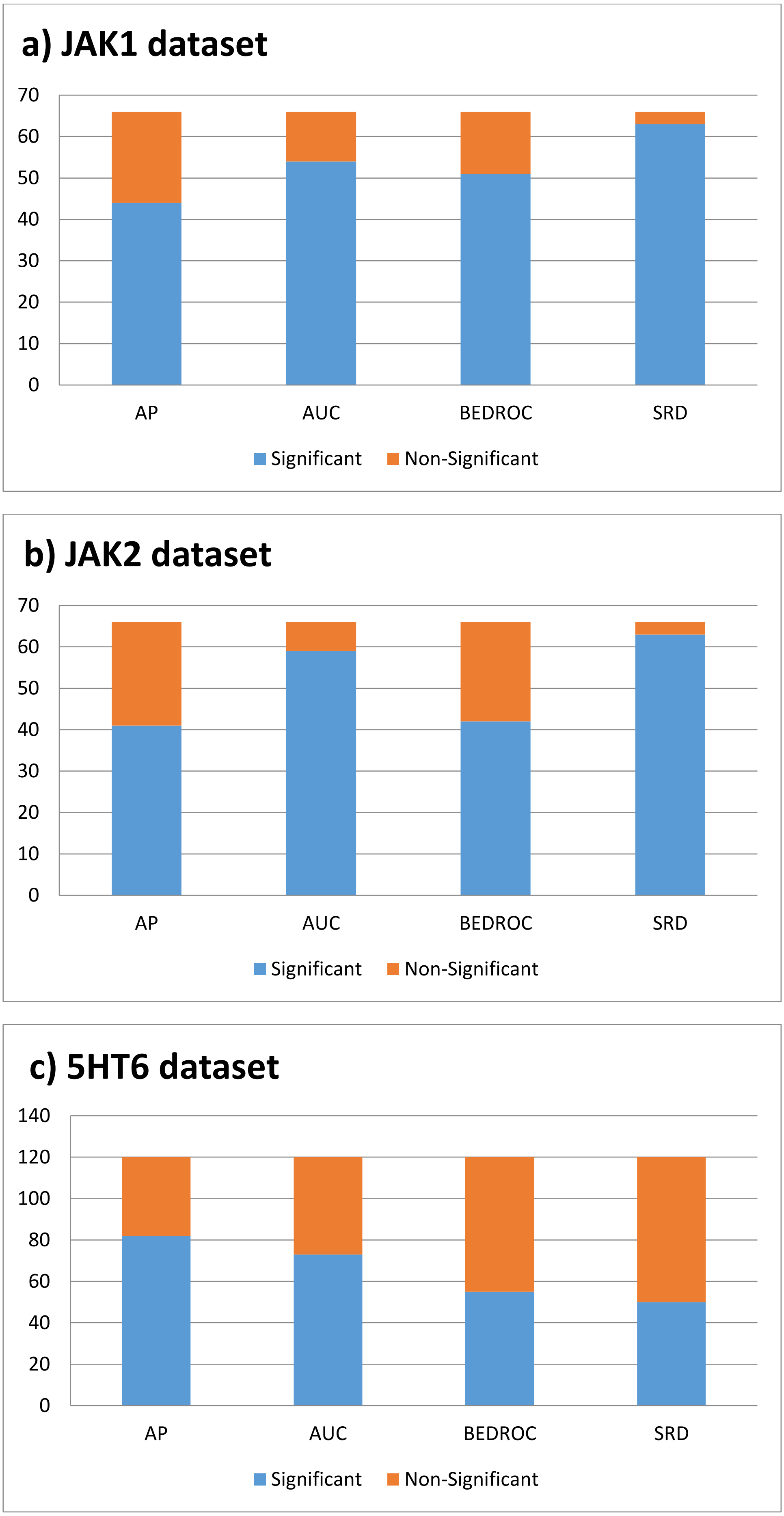

a) JAK1 dataset
70
60
50
40
30
20
10
0
AP
AUC
BEDROC
SRD
Significant
Non-Significant
b) JAK2 dataset
70
60
50
40
30
20
10
0
AP
AUC
BEDROC
SRD
Significant
Non-Significant
c) 5HT6 dataset
140
120
100
80
60
40
20
0
AP
AUC
BEDROC
SRD
Significant
Non-Significant

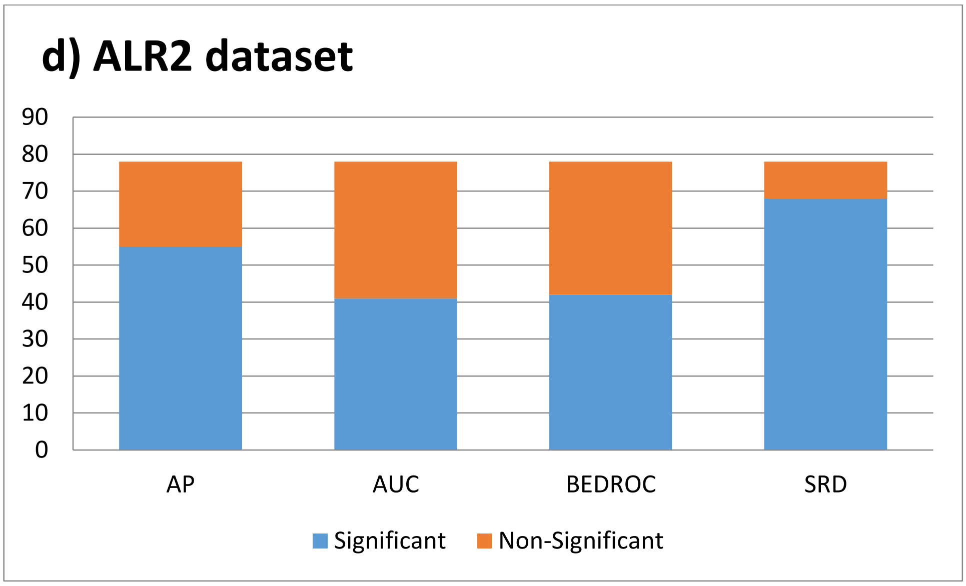

d) ALR2 dataset
90
80
70
60
50
40
30
20
10
0
AP
AUC
BEDROC
SRD
Significant
Non-Significant

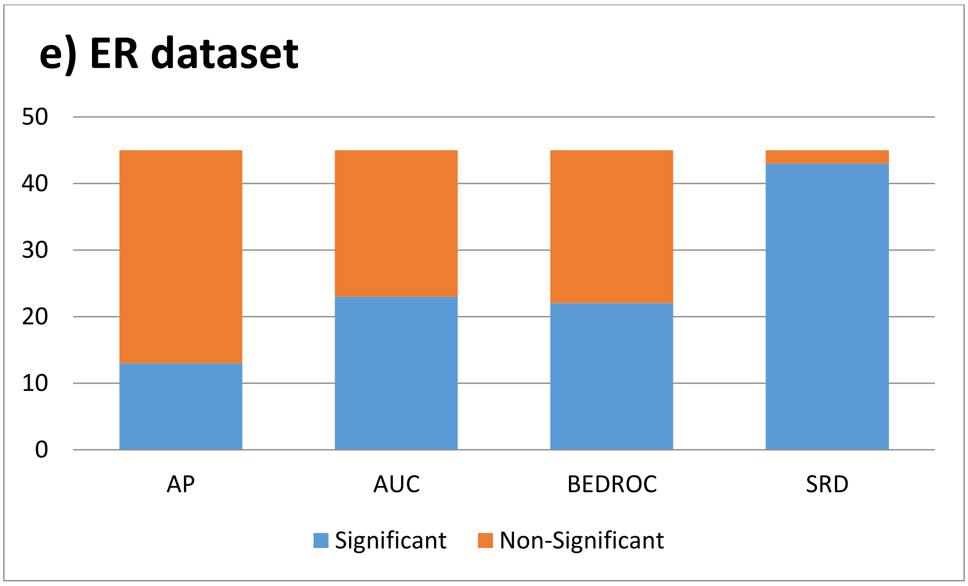

e) ER dataset
50
40
30
20
10
0
AP
AUC
BEDROC
SRD
Significant
Non-Significant

Figure S4. Result of SRD analysis for the JAK1 dataset with the use of the total number of molecules.

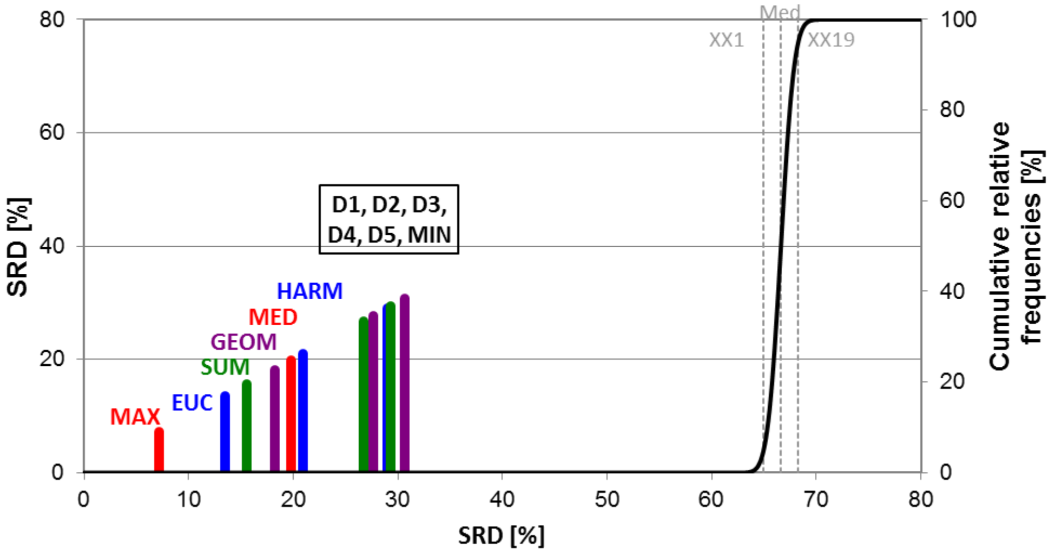

Figure S5. Comparison of the original, single-structure docking scores with the best methods based on the five case studies.

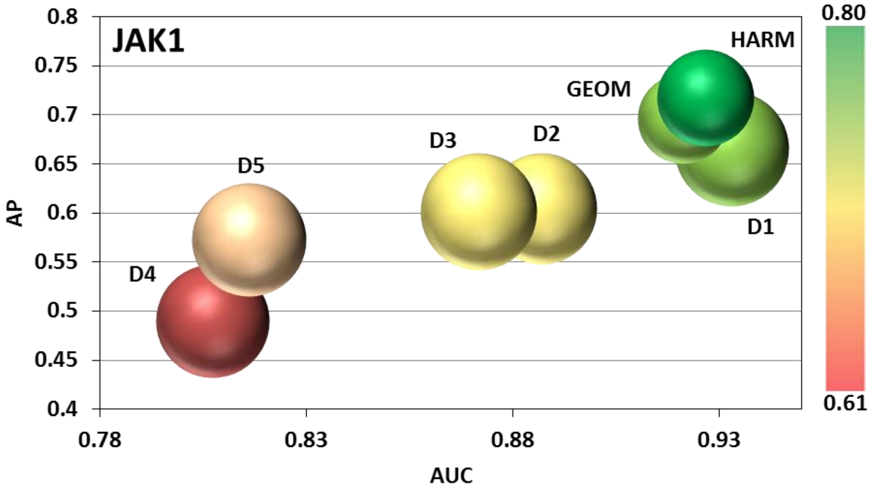

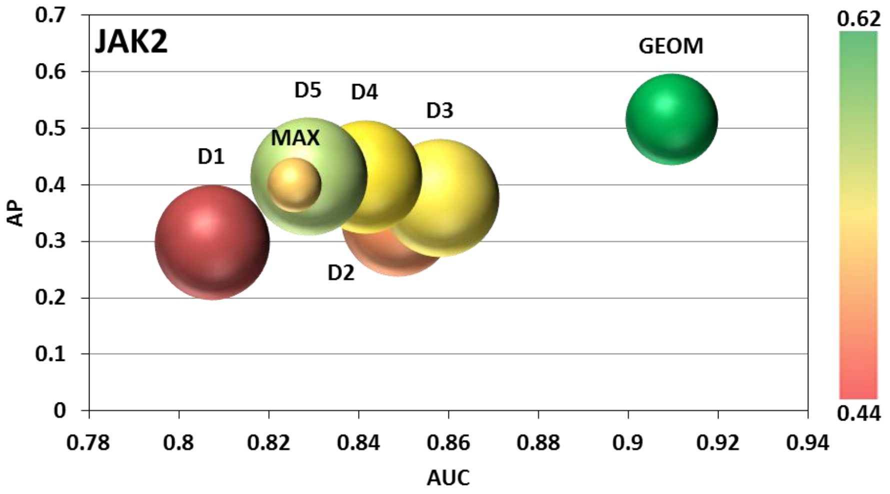

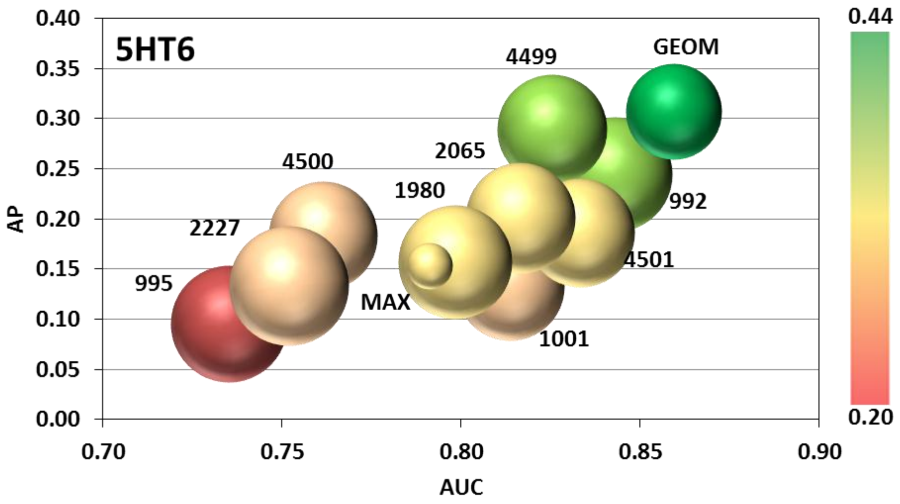
5HT6
AP
AUC
0.40
0.35
0.30
0.25
0.20
0.15
0.10
0.05
0.00
0.70
0.75
0.80
0.85
0.90
4499
GEOM
4500
2065
1980
992
2227
4501
995
MAX
1001
0.44
0.20

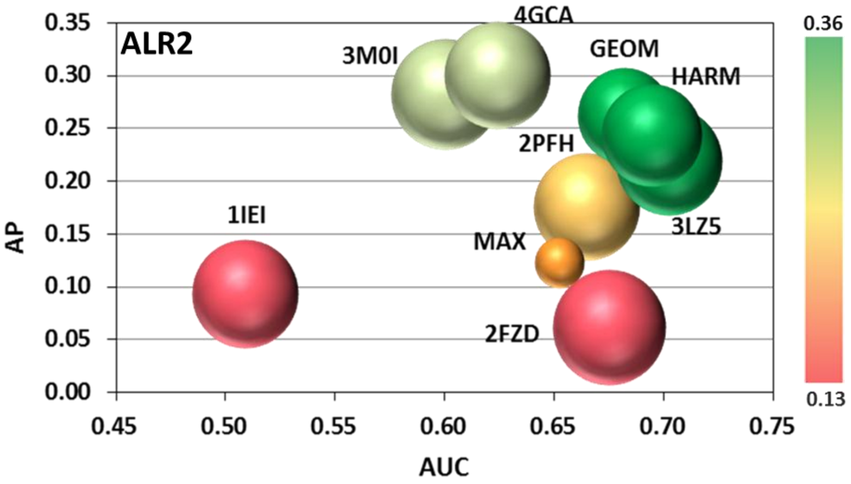
ALR2
AP
AUC
0.35
0.30
0.25
0.20
0.15
0.10
0.05
0.00
0.45
0.50
0.55
0.60
0.65
0.70
0.75
4GCA
3M0I
GEOM
HARM
2PFH
1IEI
3LZ5
MAX
2FZD
0.36
0.13

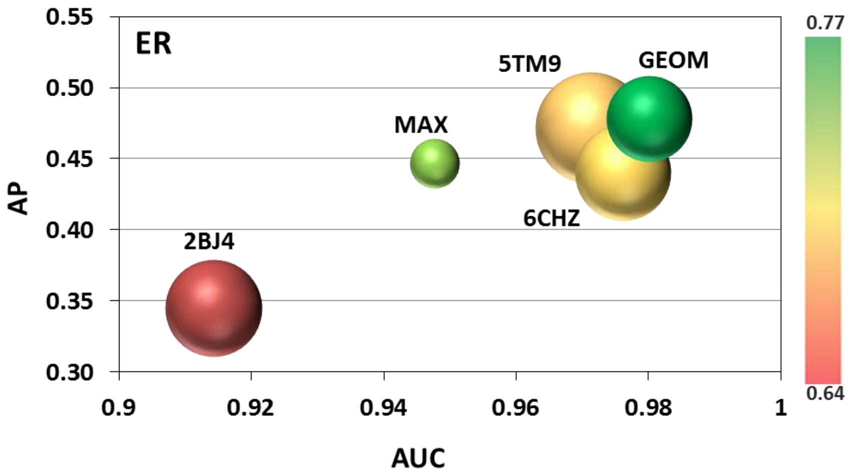
ER
AP
AUC
0.55
0.50
0.45
0.40
0.35
0.30
0.9
0.92
0.94
0.96
0.98
1
5TM9
GEOM
MAX
6CHZ
2BJ4
0.77
0.64
